# Fusion of various optimisation based feature smoothing methods for wearable and non‐invasive blood glucose estimation

**DOI:** 10.1049/syb2.12063

**Published:** 2023-03-31

**Authors:** Yiting Wei, Bingo Wing‐Kuen Ling, Danni Chen, Yuheng Dai, Qing Liu

**Affiliations:** ^1^ Faculty of Information Engineering Guangdong University of Technology Guangzhou China

**Keywords:** biomedical measurement, filtering theory, optimisation, photoplethysmography

## Abstract

The traditional blood glucose estimation method requires to take the invasive measurements several times a day. Therefore, it has a high infection risk and the users are suffered from the pain. Moreover, the long term consumable cost is high. Recently, the wearable and non‐invasive blood glucose estimation approach has been proposed. However, due to the unreliability of the acquisition device, the presence of the noise and the variations of the acquisition environments, the obtained features and the reference blood glucose values are highly unreliable. Moreover, different subjects have different responses of the infrared light to the blood glucose. To address this issue, a polynomial fitting approach to smooth the obtained features or the reference blood glucose values has been proposed. In particular, the design of the coefficients in the polynomial is formulated as the various optimisation problems. First, the blood glucose values are estimated based on the individual optimisation approaches. Second, the absolute difference values between the estimated blood glucose values and the actual blood glucose values based on each optimisation approach are computed. Third, these absolute difference values for each optimisation approach are sorted in the ascending order. Fourth, for each sorted blood glucose value, the optimisation method corresponding to the minimum absolute difference value is selected. Fifth, the accumulate probability of each selected optimisation method is computed. If the accumulate probability of any selected optimisation method at a point is greater than a threshold value, then the accumulate probabilities of these three selected optimisation methods at that point are reset to zero. A range of the sorted blood glucose values are defined as that with the corresponding boundaries points being the previous reset point and this reset point. Hence, after performing the above procedures for all the sorted reference blood glucose values in the validation set, the regions of the sorted reference blood glucose values and the corresponding optimisation methods in these regions are determined. It is worth noting that the conventional lowpass denoising method was performed in the signal domain (either in the time domain or in the frequency domain), while the authors’ proposed method is performed in the feature space or the reference blood glucose space. Hence, the authors’ proposed method can further improve the reliability of the obtained feature values or the reference blood glucose values so as to improve the accuracy of the blood glucose estimation. Moreover, the individual modelling regression method has been employed here to suppress the effects of different users having different responses of the infrared light to the blood glucose values. The computer numerical simulation results show that the authors’ proposed method yields the mean absolute relative deviation (MARD) at 0.0930 and the percentage of the test data falling in the zone A of the Clarke error grid at 94.1176%.

## Graphical abstract

The PPGs are acquired. After performing the SSA bit plane denoising and extracting various features, the feature smoothing via the fusion of various optimisation approaches is performed. Finally, the random forest is employed for performing the blood glucose estimation.

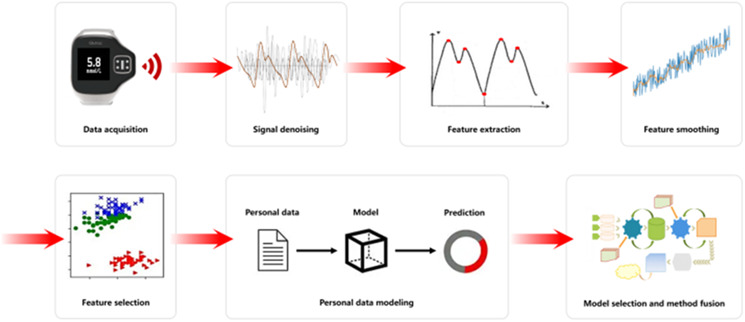

## 1 INTRODUCTION

The diabetes mellitus is a type of the metabolic diseases characterised by the hyperglycemia. It is a common disease that severely affected the human life and the public health. According to the report issued by the International Diabetes Federation (IDF), an extra of 73.6 millions of people in the worldwide will be suffered from the diabetes at 2045 [1, 2]. As the patients with the diabetes require to take the blood glucose lowering drugs or to inject the insulin to the body for avoiding the occurrence of the diabetes complications, monitoring the blood glucose values is of the great importance to the diabetes.

To monitor the blood glucose values, the traditional method is to have the measurements via the finger prickle approach several times a day. However, this approach has the infection risk [3] and the patients are suffered from the pain. Also, the long term consumable cost is very high. Therefore, there is an urgent need for developing the wearable and non‐invasive blood glucose estimation methods [4]. To perform the wearable and non‐invasive blood glucose estimation, the optical method is the commonest one among all the methods. This is because this approach allows the continuous data acquisition. It is worth noting that this approach is based on the absorption of the photons by the glucose molecules [5]. Among the whole electromagnetic spectrum, the near infrared spectroscopy shows a good correlation between the blood glucose values and the parameters in the measured photonic response [6, 7, 8]. However, as different subjects have different skin colours, different thicknesses of the fat tissue under the skin, different locations of the blood vessels, different human genes responsible for regulating the blood glucose and different dietary habits, different subjects have different responses of the infrared light to the blood glucose values. Also, since the glucose concentration in the blood is very low, the extracted features are not very sensitive to the blood glucose values. Moreover, there are many substances in the blood vessel. The absorptions of the infrared light by these substances would cause the interferences to the obtained features [9, 10, 11]. Furthermore, there are the variations in the contact pressure and the contact position exerted on the sensor during the data acquisition. In addition, there is an uncertainty in the acquisition device such as the variations of the component values in the device. Besides, the noise is contaminated to the acquired signals. Apart from that, there are some variations in the acquisition environment such as the variation of the environmental light intensity. From here, it can be concluded that there are two major factors affecting the estimation accuracy. The first one is the variations of the subjects, the devices, the environments and the acquisition conditions. The second one is the interferences from other substances in the blood and the noises. These two types of factors result to the highly unreliable obtained features and the measured reference blood glucose values [12]. Nevertheless, there is no well recognized strategy for addressing these issues in this field.

In many practical data acquisition applications, the regression based smoothing techniques including the linear regression method, the polynomial regression method, the radial basis function regression method and the Fourier basis regression method are used for performing the data pre‐processing to eliminate the acquisition errors. Since the order of the polynomial can be changed easily to meet the required specification on the accuracy, this method has a great flexibility. Therefore, the polynomial regression method was widely used in the image processing community, the data processing community and the parameter estimation community. Besides, some parameter design methods were proposed recently. For examples, the full vector based finite element method with an anisotropic perfect matched layer added to the designed model as a boundary condition was employed [13, 14, 15]. Also, the shooting method (SM) for finding the numerical solutions of the boundary value problems was employed [16]. Moreover, a sign based proportionate affine projection algorithm and a two blocked sparse memory based proportionate affine projection algorithm [17] were used for performing a fast recursive filtering used in the block sparse identification. Furthermore, the dichotomous coordinate descent iteration based method [18] was proposed.

On the other hand, the *L*_1_ norm of a vector is the sum of the absolute values of its elements. By minimising the *L*_1_ norm of an error vector, the majorities of the error points are with the small values. As the error vector is sparse, it yields a good estimation for the majority of the data points. Besides, the *L*_∞_ norm of a vector is the maximum absolute value of its elements. By minimising the *L*_∞_ norm of an error vector, the data points with the large errors are reduced. Recently, the sparse optimisation approach and the risk optimisation approach were used for performing the data smoothing [19]. Apart from that, the *L*_2_ norm of a vector is the square root of the sum of the squares of its elements. The *L*_2_ norm error values are between the *L*_1_ norm error values and the *L*_∞_ norm error values. Moreover, as the *L*_2_ norm error function is differentiable, the conventional gradient descent approach can be used to find the solution of the optimisation problem. Because of this reason, the *L*_2_ norm error function is widely used for finding the coefficients of the polynomial for performing the smoothing operation. This is also known as the least squares approach. By applying different approaches with different error norm functions for performing the smoothing operation, different smoothed features are yielded. However, no individual approach is appropriate for estimating all the data points. Hence, the fusion of these three approaches is required. Nevertheless, there is no simple rule for performing the fusion. Hence, this paper is to address this issue.

This paper is to address the unreliability of both the extracted features and the reference blood glucose values. First, this paper employs the individual modelling approach to train the regression system. This can eliminate the effects of different subjects having different responses of the infrared light to the blood glucose values. Second, this paper proposes a method to fuse the various optimisation approaches together for performing the polynomial fitting to smooth the features. This can improve the reliability of the extracted features and the reference blood glucose values. Compared to the commonly used data smoothing methods such as the moving averaging method, the Gaussian filtering method, the median filtering method, the locally weighted regression method and the Savitzky Golay (SG) filtering method, the computer numerical simulation results show that our proposed method outperforms the existing methods.

The main contributions of this paper are as follows:

- A feature domain based smoothing method is proposed to improve the reliability of the obtained features and the reference blood glucose values.
- A method is proposed for fusing the various optimisation approaches for performing the polynomial fitting to smooth the obtained features and the reference blood glucose values.
- An individual data modelling approach is proposed for performing the blood glucose estimation. This can effectively eliminate the effects of different individuals having different responses of the infrared light to the blood glucose values so as to improve the reliability of the obtained features and the reference blood glucose values.

The outline of this paper is as follows. Section 2 presents our proposed method. Section 3 presents the computer numerical simulation results. Finally, the conclusion is drawn in Section 4.

## 2 OUR PROPOSED METHOD

### 2.1 Working principle

**FIGURE 1:**
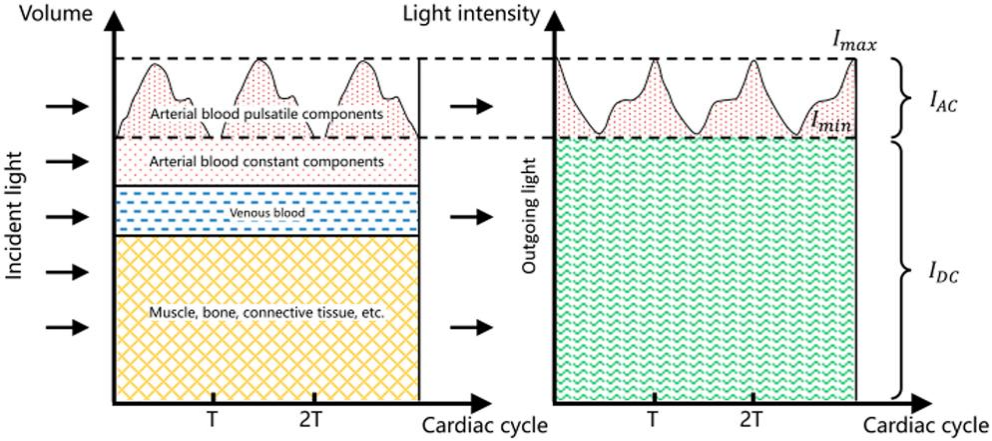
The direct current (DC) component and the alternating current (AC) component of the PPG due to the different absorptions of the near infrared light by the different substances.

The near infrared spectroscopy is a study of the electromagnetic wave. When a beam of photons is hit on the substances in the human tissue [7, 8], the photons are partially absorbed and refracted as well as partially reflected and scattered. Then, the received optical signal is converted to an electrical signal and it is amplified. The electrical signal is called the photoplethysmogram (PPG). It is worth noting that there is a linear correlation between the concentration of a particular substance in the human tissue and the parameters in the near infrared signal. These parameters are related to the spectral absorption. Therefore, the concentrations of the substances in the human tissue can be estimated by extracting the near infrared spectral absorption coefficients from the PPGs [20]. In fact, the non‐invasive blood glucose estimation is a typical example of applying the near infrared spectroscopy to the human physiology [6]. However, the human tissue consists of the various substances. In fact, different substances have different ratios of the total number of photons being absorbed to that being reflected for a given beam of photons with a particular wavelength hitting on the human tissue. In particular, for the glucose molecule, the maximum ratio of the total number of photons being absorbed to that being reflected is occurred at the beam of photons with a wavelength being equal to 1600 nm [21]. Since the volumes of the skin, the muscle and the bone do not change during the entire circulation cycle of the heartbeat, the absorptions of the optical light due to these substances are constant. On the other hand, the volume of the blood flowing in the artery increases and decreases quasi‐periodically during the entire circulation cycle of the heartbeat. Hence, the reflected near infrared light received by the photoelectric receiver also exhibits a quasi‐periodical pattern. As a result, the obtained PPG can be represented as the sum of the direct current (DC) component and the alternating current (AC) component. By extracting the AC component out, the characteristics of the blood flow can be characterised. Figure 1 shows the DC component and the AC component of the PPG due to the absorptions of the near infrared light by all the substances. To further investigate this phenomenon, please refer to the Lambert Beer law.

**FIGURE 2:**
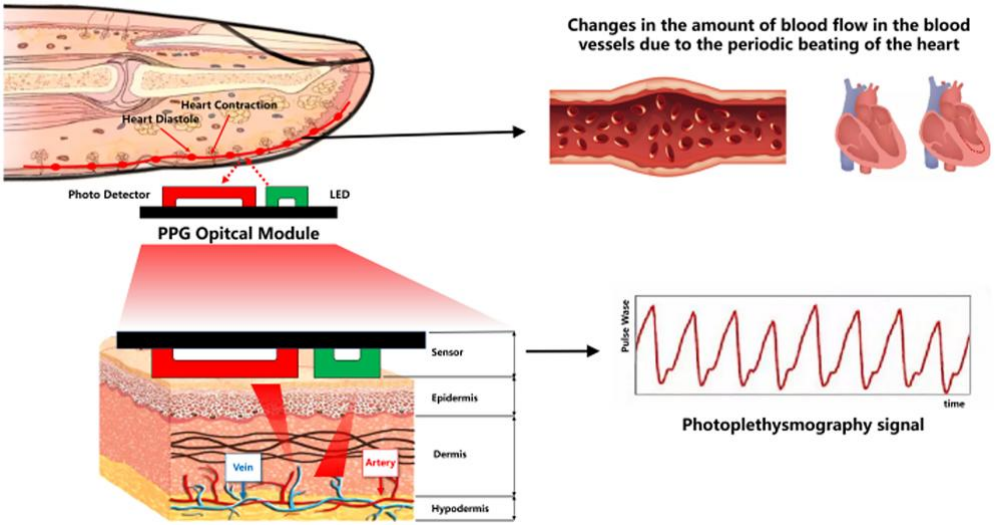
A diagram showing how the PPG is related to the blood flow in the finger.

It is worth noting that the autonomic nerves play a vital role in regulating the various physiological activities of the body [22]. However, different diabetic patients have different degrees of the damage in the cardiac autonomic nerves. Hence, the patients with the hyperglycemia have the various degrees of the arrhythmia [23]. To quantify the arrhythmia, the heart rate variability (HRV) is employed. It refers to the variations of the heart rates (HRs). Therefore, the variations of the HRs due to the loss of the regulations of the various physiological activities because of the damage in the cardiac autonomic nerves [24, 25] can be used to estimate the blood glucose levels. To compute the HRV, the conventional algorithms for detecting the QRS points were employed for locating the R waves in the acquired electrocardiograms (ECGs) [26]. Then, the primary method based on generating the velocity maps of the HRV was employed to compute the HRV. Finally, the features derived from the obtained HRV were used to estimate the arterial blood glucose levels [27]. However, as the device for acquiring the ECGs is required to place away from the heart, the blood flows at these positions are relatively slow. Also, the acquisition device has to touch the skin tightly to acquire the ECGs. Nevertheless, the loss of contact is usually happened and it results to the unreliable acquired ECGs. Moreover, the interference will degrade the quality of the acquired ECGs. Hence, it is challenging to estimate the blood glucose level via the acquired ECGs from a practice viewpoint. To address the above difficulty, a more practical method is required for computing the HRV. In particular, the PPG is employed. Figure 2 shows how the PPG is related to the blood flow in the finger. A large number of experimental data shows that the HR estimated by the PPGs is consistent with that estimated by the ECGs when the subjects are in the relaxed state [28]. That is, the interval between the two consecutive peaks (PP interval) estimated by the PPGs is very close to the RR interval estimated by the ECGs. Moreover, the nonlinear dynamics of the PPGs are very close to that of the ECGs. Hence, the HRV can be derived using the PPGs. As the PPGs can be acquired more easily [29, 30, 31], this paper only employs the PPGs to perform non‐invasive blood glucose estimation.

Besides using the absorption of the near infrared light and the HRV to estimate the blood glucose levels, the transit time of the PPG was also used to estimate the blood glucose values [32]. In particular, the machine learning approach is used to model the relationship between the transit time of the PPG and the blood glucose values. However, finding the transit time of the PPG is challenging if the synchronous ECG is absence.

### 2.2 Dataset

**FIGURE 3:**
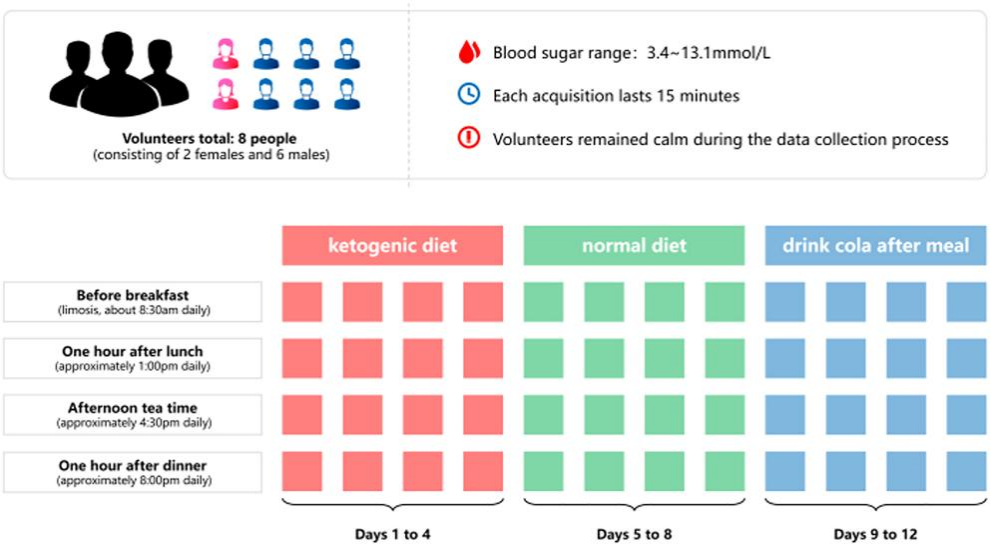
The arrangement of taking three different types of diets.

Eight volunteers are recruited for performing the data acquisition. In particular, the subjects take the ketogenic diet during the first 4 days, the normal diet between the fifth day and the eighth day as well as drinking cola after lunch and dinner during the last 4 days. Figure 3 shows the arrangement of taking these three different types of diets. It is worth noting that taking these three different types of diets will result to the very different blood glucose levels.

### 2.3 Our proposed algorithm

**FIGURE 4:**
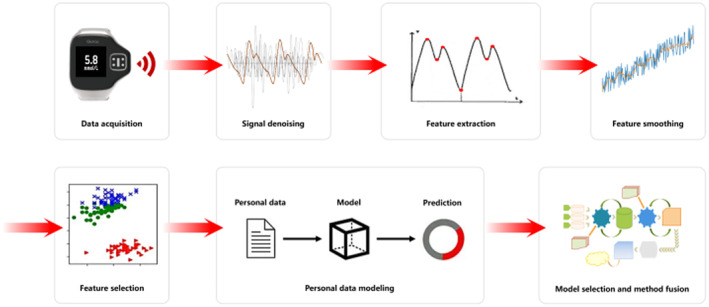
The block diagram of our proposed method.

The block diagram of our proposed algorithm is shown in Figure 4. First, the PPGs are acquired. Second, the acquired PPGs are divided into the training set and the test set. Third, all the PPGs in both the training set and the test set are denoised. Fourth, the features are extracted from each denoised PPG in both the training set and the test set. Fifth, the extracted features or the reference blood glucose values in the training set are smoothed based on the method proposed in this paper. Sixth, some smoothed features are selected. Then, the new lower dimensional feature vectors in both the training set and the test set are formed. Finally, the regression models are built using the new feature vectors and the reference blood glucose values in the training set. Here, the individual modelling approach is employed.

#### 2.3.1 Acquiring the PPGs as well as taking the reference blood glucose values and the reference blood pressure values

**FIGURE 5:**
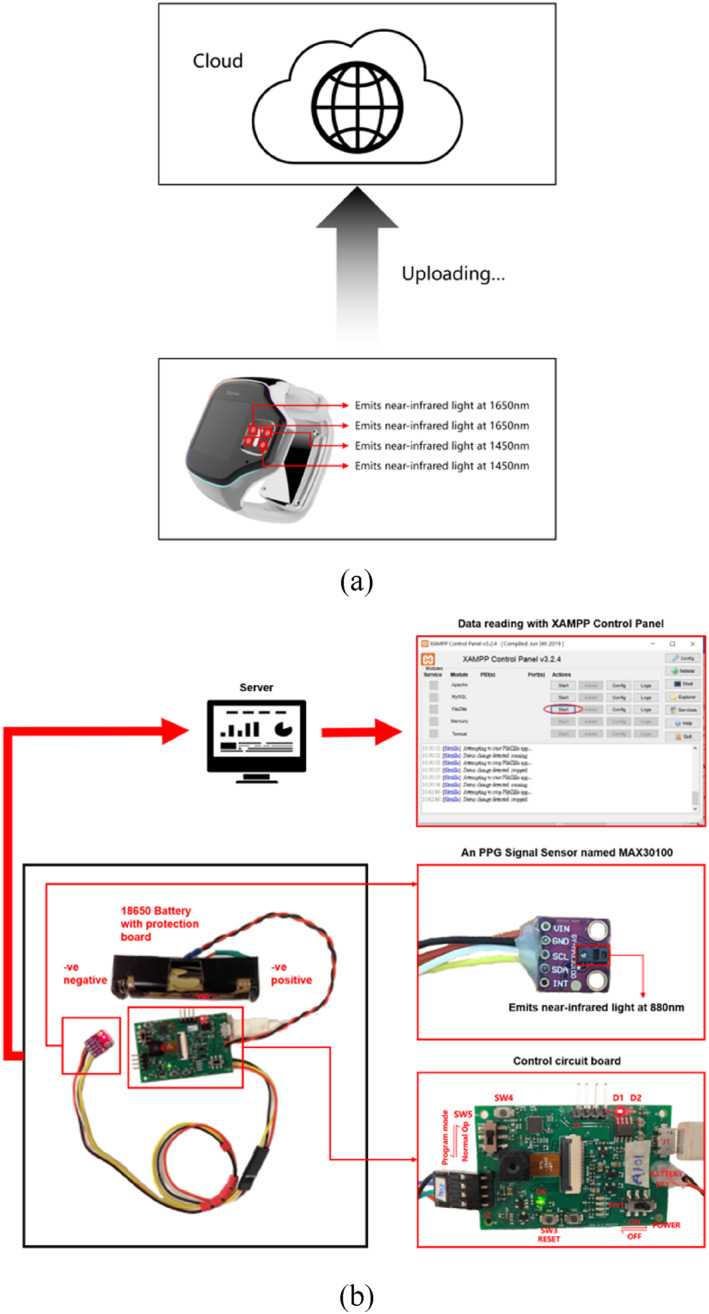
(a) The first acquisition device emitting the lights with their wavelengths being equal to 1450 and 1650 nm (b) The second acquisition device emitting the light with its wavelength being equal to 880 nm.

There are two sets of acquisition devices for acquiring the PPGs. Figure 5 shows these two sets of the acquisition devices. The wavelength of the near infrared light emitted by the first acquisition device is 880 nm operating the sampling rate at the 1000 Hz. The wavelengths of the near infrared lights emitted by the second set of the acquisition devices are 1450 and 1650 nm operating the sampling rate at the 50 Hz. As discussed in Section IIA, both the DC part and the AC part of the PPGs as well as the HRV can be used to estimate the blood glucose level. Here, the acquired PPGs based on the LED emitting the light with the wavelength being equal to 880 nm are used to compute the HRV, while the AC part and the DC part of the acquired PPGs based on the LEDs emitting the lights with their wavelengths being equal to 1450 and 1650 nm are used to extract the features related to the absorption of the photons due to the blood glucose.

The Bene Check blood glucose metre and the Yuwell sphygmomanometer are employed to take the reference blood glucose value and the reference blood pressure values, respectively. Both devices have the FDA certification. Here, three measurements are taken every time and every measurement only takes one reference blood glucose value as well as one systolic blood pressure (SBP) value and one diastolic blood pressure (DBP) value. Then, the average of these three reference blood glucose values as well as the average of these three SBP values and the average of these three DBP values are employed as the final reference values.

In order to obtain the reference blood glucose values and the reference blood pressure values, the Bene Check blood glucose metre and the Yuwell sphygmomanometer are employed for the acquisitions, respectively. It is worth noting that both the devices have the corresponding FDA certifications. In this paper, three measurements are taken every time and every measurement only takes one reference blood glucose value as well as one SBP value and one diastolic blood pressure (DBP) value. Then, the average of these three reference blood glucose values as well as the average of these three SBP values and the average of these three DBP values are employed as the corresponding final reference values.

#### 2.3.2 Definition of the training set and the test set

**FIGURE 6:**
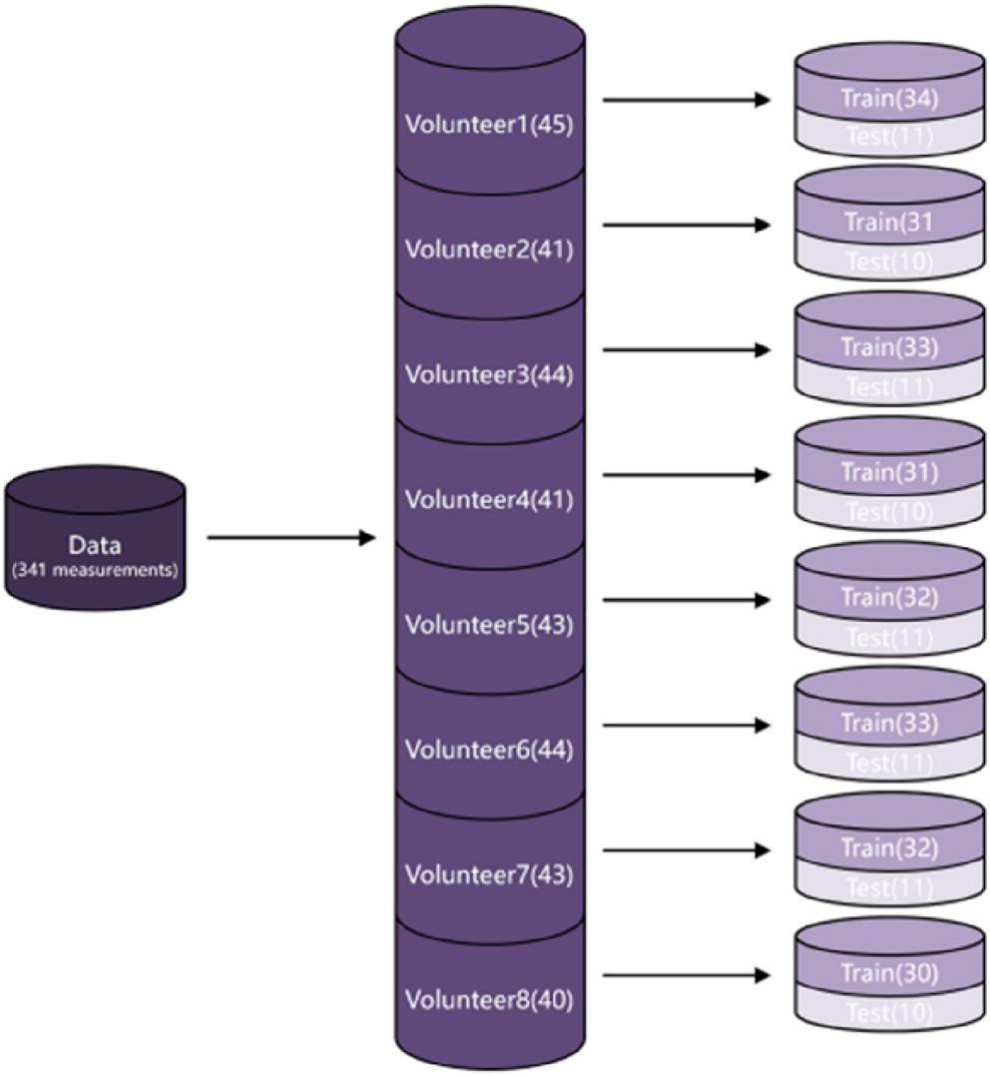
The exact total numbers of the PPGs in both the training set and the test set based on the various individual subjects.

To perform the training, all the PPGs acquired from an individual subject are mixed together. Then, the individual dataset is randomly divided into two non‐overlapped subsets. They are the training set and the test set. In particular, approximately 75% of the individual dataset is defined as the training set and the rest approximately 25% of the individual dataset is defined as the test set. Figure 6 shows the exact total numbers of the PPGs in both the training set and the test set based on an individual subject.

#### 2.3.3 Denoising

**FIGURE 7:**
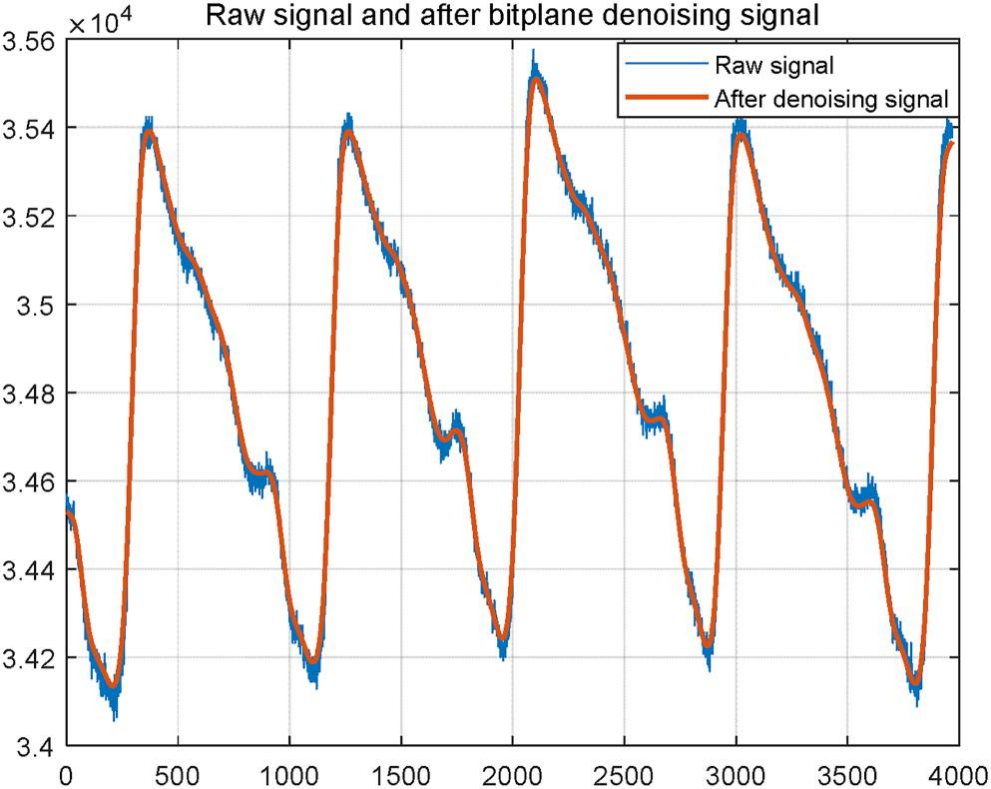
A realization of the raw PPG and the corresponding denoised PPG.

Since the HRs of different subjects are different, the frequency bands of different PPGs acquired from different subjects are different. Hence, an adaptive based denoising method is required to address this problem. Among them, the singular spectrum analysis (SSA) based denoising method is the most common adaptive denoising method. However, the conventional SSA based denoising method is to discard the whole SSA component. In this case, some signal information is lost. To address this issue, an SSA based bit plane method is employed for denoising the PPGs. First, the SSA is applied to all the PPGs in both the training set and the test set. Here, the SSA decomposes each PPG into 32 components. Second, for each value in each SSA component, it is represented using a finite number of bits. In this paper, the word length of each value is 13 bits. Third, the noise level of each SSA component is estimated. Fourth, the total number of bits corresponding to the noise contaminated to each SSA component is set as the logarithm (based two) of its estimated noise level. Fifth, these noise bits are set to zero. Sixth, each value in each SSA component is reconstructed using the retained bits. Seventh, each PPG is reconstructed by summing up all the processed SSA components together. Eighth, the SSA is performed again on each reconstructed PPG. Ninth, only the first SSA component is retained and it is taken as the denoised PPG. Figure 7 shows a realization of the raw PPG and the corresponding denoised PPG. It can be seen from Figure 7 that the noise is significantly suppressed after performing the denoising.

#### 2.3.4 Feature extraction

103 features are extracted in each denoised PPG in both the training set and the test set. These 103 features are classified into five categories.

Category 1 (Features related to the meal time): First, each meal time and each data acquisition time are recorded in each measurement. Here, all the times are defined as the total number of hours after the midnight of that day. Then, the difference between the data acquisition time and the previous meal time is computed. This difference is taken as the feature. Therefore, there is only one feature for each feature vector in this category.

Category 2 (Features related to the blood pressure): Both the SBP value and the DBP value are employed as the features. Therefore, there are two features for this category.

Category 3 (Features related to the absorption of the infrared light by the blood glucose): There are two LEDs emitting the light with the wavelength being equal to 1450 nm and another two LEDs emitting the light with the wavelength being equal to 1650 nm. Therefore, there are four PPGs acquired at each time. Since the AC component and the DC component of the acquired PPGs are related to the absorption of the infrared light by the blood glucose, the mean and the variance of each PPG are computed and they are used as the features. As a result, there are eight features for each feature vector in this category.

Category 4 (Features related to the HRV): There is a LED emitting the light with the wavelength being equal to 880 nm. Here, there are four finger cots covering the LED. Hence, there are also four PPGs acquired at each measurement. For each PPG, there are 13 features related to the HRV with six features extracted from the time domain and seven features extracted from the frequency domain. For those six features extracted from the time domain, they are the average and the standard deviation (SD) of the PP intervals, the root mean squares and the SD of the differences between two consecutive PP intervals as well as the total number and the percentage of the total number of the differences between two consecutive PP intervals with the differences being greater than 50 ms. For those seven features extracted from the frequency domain, they are the total power of the PPG in the whole frequency spectrum, that in the frequency band between 0 and 0.04 Hz (VLF), that in the frequency band between 0.04 and 0.15 Hz (LF) and that in the frequency band between 0.15 and 0.4 Hz (HF), as well as the ratio of the LF to the HF, the normalised LF and the normalised HF. Overall, there are 52 features for each feature vector in this category.

Category 5 (Features related to the HR): The same four PPGs defined in Category 4 are employed for extracting the HR features. First, the HRs are estimated based on the PP intervals of each PPG. Then, the mean, the median, the mode, the variance, the SD, the range, the interquartile range, the skewness, the kurtosis and the median absolute deviation of the HRs are computed. They are used as the features. Here, there are ten features for each PPG. As there are four PPGs for each measurement, there are 40 features for each feature vector in this category.

#### 2.3.5 Smoothing the feature values or the reference blood glucose values

To smooth the feature values or the reference blood glucose values, first they are normalised. In particular, let ${\hat{y}}_{i}$ be the vector containing the $i^{th}$ feature extracted from all the PPGs in the training set. Then, ${\hat{y}}_{i}$ is normalised to the unit energy vector. Let $g_{i}=\frac{1}{\sqrt{{\hat{y}}_{i}^{T}{\hat{y}}_{i}}}$ be the normalised gain. Let $y_{i}$ be the normalised vector. That is, $y_{i}=g_{i}{\hat{y}}_{i}$. Likewise, for the $i^{th}$ feature extracted from a PPG in the test set, the feature value is multiplied to $g_{i}$ to obtain the normalised feature value.

Second, a polynomial fitting approach is employed for smoothing the features or the reference blood glucose values in the training set. It is worth noting that a large value of the polynomial order does not achieve the smoothing purpose. On the other hand, a small value of the polynomial order results to the over smoothing. Hence, this paper sets the order of the polynomial equal to 3. Moreover, three different formulations are employed for finding the coefficients of the polynomial. They are based on the *L*_2_ norm optimisation method, the *L*_1_ norm optimisation method and the *L*_∞_ norm optimisation method. Let N be the total number of the measurements in the training set. For each normalised feature, the normalised feature values and the reference blood glucose values are first sorted according to the ascending order of the reference blood glucose values. Let $\left(x_{i},y_{i}\right)$ be the $i^{\text{th}}$ sorted pair of the normalised feature value and the corresponding reference blood glucose value. Let $a_{i}$ be the coefficients of the polynomial.

##### Smoothing the reference blood glucose values via the *L*_2_ norm optimisation method

Let

(1a) $$X=\left[\begin{array}{cccc} 1 & x_{1} & \text{⋯} & x_{1}^{3}\\ 1 & x_{2} & \text{⋯} & x_{2}^{3}\\ \text{⋮} & \text{⋮} & \text{⋱} & \text{⋮}\\ 1 & x_{N} & \text{⋯} & x_{N}^{3} \end{array}\right],$$

(1b) $$a=\left[\begin{array}{c} a_{0}\\ \text{⋮}\\ a_{3} \end{array}\right]$$

and

(1c) $$y=\left[\begin{array}{c} y_{1}\\ \text{⋮}\\ y_{N} \end{array}\right].$$

Let $J_{2}\left(a\right)$ be the objective function of the optimisation problem. Here, it is defined as the energy of the error between the estimated blood glucose values and the actual blood glucose values. In particular, the optimisation problem is formulated as follows:

(1d) $$\min_{a}J_{2}\left(a\right)=\sum_{i=1}^{N}{\left(a_{0}+a_{1}x_{i}+\text{…}+a_{3}x_{i}^{3}-y_{i}\right)}^{2}.$$

This is equivalent to

(1e) $$\min_{a}J_{2}\left(a\right)={\left\|Xa-y\right\|}^{2}.$$

By computing its gradient vector with respect to a and setting its gradient vector to the zero vector, we have the following equation:

(1f) $$a={\left(X^{T}X\right)}^{-1}X^{T}y.$$

Once a is found, y is replaced by Xa. This is the vector of the smoothed reference blood glucose values. The above procedures are repeated for each feature and the mean of these vectors of the smoothed reference blood glucose values are taken as the final vector of the smoothed reference blood glucose values.

##### Smoothing the reference blood glucose values via the *L*_1_ norm optimisation method

Let $J_{1}\left(a\right)$ be the objective function of the optimisation problem. Here, it is defined as the sum of the absolute error between the estimated blood glucose values and the actual blood glucose values. In particular, the optimisation problem is defined as follows:

(2a) $$\min_{a}J_{1}\left(a\right)=\sum_{i=1}^{N}\left|a_{0}+a_{1}x_{i}+\text{…}+a_{3}x_{i}^{3}-y_{i}\right|.$$

This is equivalent to

(2b) $$\min_{a}J_{1}\left(a\right)={\left\|Xa-y\right\|}_{1}.$$

Let z be a dummy vector and let ι be a vector with all its elements being equal to one. The above optimisation problem is equivalent to the following optimisation problem:

(2c) $$\min_{\left(a,z\right)}\iota^{T}z,$$

subject to Xa−y≤z,

and −Xa+y≤z.

Let $I_{N}$ be the N×N identity matrix. Let $\tilde{a}=\left[\begin{array}{c} a\\ z \end{array}\right]$, $f=\left[\begin{array}{c} 0\\ \iota \end{array}\right]$, $\tilde{X}=\left[\begin{array}{cc} X & -I_{N}\\ -X & -I_{N} \end{array}\right]$ and $\tilde{y}=\left[\begin{array}{c} y\\ -y \end{array}\right]$. The above optimisation problem becomes the following standard linear programing problem:

(2d) $$\min_{\tilde{a}}f^{T}\tilde{a},$$

subject to $\tilde{X}\tilde{a}\leq \tilde{y}$.

The solution of this standard linear programing problem can be found via the simplex method. By extracting the first four elements in $\tilde{a}$ and multiplying X in front of this extracted vector, the vector of the smoothed reference blood glucose values is obtained. Likewise, y is replaced by this smoothed reference blood glucose values and the above procedures are repeated for each feature. Finally, the mean of these vectors of the smoothed reference blood glucose values are taken as the final vector of the smoothed reference blood glucose values.

##### Smoothing the reference blood glucose values via the *L*_∞_ norm optimisation method

Let $J_{\infty }\left(a\right)$ be the objective function of the optimisation problem. Here, it is defined as the maximum absolute error between the estimated blood glucose values and the actual blood glucose values. In particular, the optimisation problem is defined as follows:

(3a) $$\min_{a}J_{\infty }\left(a\right)=\max_{i}\left|a_{0}+a_{1}x_{i}+\text{…}+a_{3}x_{i}^{3}-y_{i}\right|.$$

This is equivalent to

(3b) $$\min_{a}J_{\infty }\left(a\right)={\left\|Xa-y\right\|}_{\infty }.$$

Let ε be a dummy scalar. The above optimisation problem is equivalent to the following optimisation problem:

(3c) $$\min_{\left(a,\varepsilon \right)}\varepsilon ,$$

subject to Xa−y≤ει,

and −Xa+y≤ει.

Let $\hat{a}=\left[\begin{array}{c} a\\ \varepsilon \end{array}\right]$, $\hat{f}=\left[\begin{array}{c} 0\\ 1 \end{array}\right]$ and $\hat{X}=\left[\begin{array}{cc} X & -\iota \\ -X & -\iota \end{array}\right]$. The above optimisation problem becomes the following standard linear programing problem:

(3d) $$\min_{\hat{a}}{\hat{f}}^{T}\hat{a},$$

subject to $\hat{X}\hat{a}\leq \tilde{y}$.

Likewise, the solution of this standard linear programing problem can be found via the simplex method. Then, by extracting the first four elements in $\hat{a}$ and multiplying X in front of this extracted vector, the vector of the smoothed reference blood glucose values is obtained. Next, y is replaced by this smoothed reference blood glucose values and the above procedures are repeated for each feature. Finally, the mean of these vectors of the smoothed reference blood glucose values are taken as the final vector of the smoothed reference blood glucose values.

##### Smoothing the feature values

To smooth the feature values, the above three methods are repeated by swapping the vector of the feature values and the vector of the reference blood glucose values. However, instead of iterating the above procedures for each feature in the above algorithms, now it is only require to perform one iteration in the above algorithms because there is only one vector of the reference blood glucose values.

#### 2.3.6 Feature selection

In this paper, the random forest (RF) is employed to rank the importance of the features. The features with the highest 25 importance are selected. Hence, the new training set and the new test set with 25 dimensional feature vectors are formed. It is worth noting that the individual modelling approach is employed. Therefore, different features are selected for different subjects.

#### 2.3.7 Regression models

To develop the regression models, three models are employed. They are the support vector regression (SVR) model, the Gaussian regression model and the RF regression model. The parameters in these three models are found using the same training set.

#### 2.3.8 Fusion of the various models

To yield a more accurate blood glucose estimation, the features or the reference blood glucose values smoothed by the polynomials with their coefficients found via the *L*_1_ norm optimisation method, the *L*_2_ norm optimisation method and the *L*_∞_ norm optimisation method are fused together. In particular, $\frac{2}{3}$ of the data in the previous training set is redefined as the new training set and the rest $\frac{1}{3}$ of the data in the previous training set is defined as the validation set. Therefore, 50% of the overall data is used to form the new training set, 25% of the overall data is used to form the validation set and the rest 25% of the overall data is used to form the test set. First, a RF based regression model is built using the feature vectors and the reference blood glucose values in the new training set with their values smoothed by the polynomial found via the *L*_1_ norm optimisation method. Then, the built model is applied to the feature vectors in the validation set for performing the blood glucose estimation. Next, the absolute differences between the estimated blood glucose values and the reference blood glucose values in the validation set are computed. After that, these absolute difference values are sorted according to the ascending order of the reference blood glucose values in the validation set. Let $e_{1}$ be the vector of these sorted absolute difference values. Likewise, the above procedures are repeated for the features or the reference blood glucose values smoothed by the polynomials found via the *L*_2_ norm optimisation method and the *L*_∞_ norm optimisation method. Let $e_{2}$ and $e_{\infty }$ be the vectors of these sorted absolute difference values, respectively. Now, for each reference blood glucose value in the validation set, there are three absolute difference values based on the polynomials found by the *L*_1_ norm optimisation method, the *L*_2_ norm optimisation method and the *L*_∞_ norm optimisation method. The optimisation method corresponding to the minimum value among these three values is selected. It is found that the selected optimisation methods can be partitioned into several regions according to the sorted reference blood glucose values. To determine the regions of the sorted reference blood glucose values and the corresponding selected optimisation method, the accumulate probability approach is proposed. That is, the accumulate probability of each selected optimisation method is computed. Let ε be a threshold value. In this paper, ε is set at 0.6. If the accumulate probability of any selected optimisation method at a point is greater than ε, then the accumulate probabilities of these three selected optimisation methods at that point are reset to zero and a range of the sorted blood glucose values are defined as that with the corresponding boundaries points being the previous reset point and this reset point. Hence, after performing the above procedures for all the reference blood glucose values in the validation set, the regions of the sorted reference blood glucose values and the corresponding optimisation methods in these regions are determined. Finally, after applying the RF models formulated using these three optimisation methods to a feature vector in the test set, there are three estimated blood glucose values. Then, the mean of these three estimated blood glucose values is computed. Next, according to the model developed in the validation set, the corresponding blood glucose value is selected as the final estimated blood glucose value.

## 3 COMPUTER NUMERICAL SIMULATION RESULTS

### 3.1 Results on various smoothing methods

As discussed in Section 2.3.5, the smoothing operations can be performed in the feature space or in the reference blood glucose space. This section determines the spaces whether the smoothing operations are performed or not via conducting the computer numerical simulations. Here, there are 10 different smoothing operations. They are only smoothing the feature values via the *L*_1_ norm optimisation method (denoted as O_f1), only smoothing the feature values via the *L*_2_ norm optimisation method (denoted as O_f2), only smoothing the feature values via the *L*_∞_ norm optimisation method (denoted as O_f∞), only smoothing the reference blood glucose values via the *L*_1_ norm optimisation method (denoted as B1_O), only smoothing the reference blood glucose values via the *L*_2_ norm optimisation method (denoted as B2_O), only smoothing the reference blood glucose values via the *L*_∞_ norm optimisation method (denoted as B∞_O), smoothing both the feature values and the reference blood glucose values via the *L*_1_ norm optimisation method (denoted as B1_f1), smoothing both the feature values and the reference blood glucose values via the *L*_2_ norm optimisation method (denoted as B2_f2), smoothing both the feature values and the reference blood glucose values via the *L*_∞_ norm optimisation method (denoted as B∞_f∞), and neither smoothing the feature values nor the reference blood glucose values (denoted as O_O).

In this paper, three different criteria are employed for evaluating the blood glucose estimation performances. They are the value of R, the mean absolute error (MAE) and the root mean squares error (RMSE). Here, the SD of the estimated blood glucose values is also presented. Table 1, Table 2 and Table 3 show the obtained performances yielded by various smoothing methods via the RF regression model, the SVR model and the Gaussian regression model, respectively. It can be seen from these tables that the RF regression model yields the best results in terms of all the above three criteria compared to the SVR model and the Gaussian regression model. Moreover, for the RF regression model, it can be seen from Table 1 that the O_f∞ method yields the best estimation result in terms of all the above three criteria. On the other hand, for both the SVR model and the Gaussian regression model, it can be seen from Table 2 and Table 3 that different methods would yield the best estimation results for different criteria. Hence, fusing different methods together would yield the better results.

**TABLE 1:** The values of R, mean absolute error (MAE) and root mean squares error (RMSE) yielded by the various smoothing methods via the random forest (RF) regression model.

Methods	R	MAE±SD (mmol/L)	RMSE (mmol/L)
O_f1	0.8959	0.9581 ± 1.4041	1.1610
O_f2	0.8743	0.8889 ± 1.5229	1.1509
O_f∞	**0.9103**	**0.7991 ± 1.8746**	**1.1319**
B1_O	0.7834	0.9669 ± 1.8230	1.1692
B2_O	0.7669	1.0080 ± 1.8225	1.1673
B∞_O	0.7699	1.0160 ± 1.9302	1.1772
B1_f1	0.7895	1.0923 ± 1.8227	1.1709
B2_f2	0.7966	1.0285 ± 1.8150	1.1685
B∞_f∞	0.7576	1.0158 ± 1.9332	1.1769
O_O	0.7545	1.1908 ± 1.9416	1.1782

*Note*: The bold values refer to the values corresponding to the best results.

**TABLE 2:** The values of R, mean absolute error (MAE) and root mean squares error (RMSE) yielded by the various smoothing methods via the support vector regression (SVR) model.

Methods	R	MAE±SD (mmol/L)	RMSE (mmol/L)
O_f1	0.7402	**0.9443 ± 1.9292**	1.1955
O_f2	**0.7917**	1.0752 ± 1.9659	1.1891
O_f∞	0.5361	2.3640 ± 2.4195	1.2745
B1_O	0.7156	1.0558 ± 1.8306	1.1879
B2_O	0.7113	1.0968 ± 1.8245	1.1867
B∞_O	0.6777	1.1017 ± 1.9304	1.1953
B1_f1	0.6622	1.1144 ± 1.8095	1.1940
B2_f2	0.7279	0.9757 ± 1.8335	1.1848
B∞_f∞	0.6197	1.2986 ± 1.8862	1.1973
O_O	0.7411	0.9978 ± 1.8770	**1.1839**

*Note*: The bold values refer to the values corresponding to the best results.

**TABLE 3:** The values of R, mean absolute error (MAE) and root mean squares error (RMSE) yielded by the various smoothing methods via the Gaussian regression model.

Methods	R	MAE±SD (mmol/L)	RMSE (mmol/L)
O_f1	0.7146	**0.9169 ± 1.9050**	1.1907
O_f2	0.6263	1.1264 ± 2.0025	1.2011
O_f∞	0.6813	1.1086 ± 1.9091	1.1965
B1_O	0.6998	1.0938 ± 1.8078	1.1897
B2_O	**0.7270**	1.0050 ± 1.8243	**1.1850**
B∞_O	0.6646	1.1179 ± 1.9403	1.1983
B1_f1	0.6822	1.1007 ± 1.8372	1.1911
B2_f2	0.6831	1.1013 ± 1.8404	1.1900
B∞_f∞	0.6523	1.1446 ± 1.9513	1.1993
O_O	0.6064	1.2252 ± 2.1098	1.2086

*Note*: The bold values refer to the values corresponding to the best results.

In order to demonstrate the effectiveness of fusing the various methods and the effectiveness of using the individual modelling approach, Table 4 shows the values of R, MAE, RMSE and MARD yielded by the fusion method, the O_f1 method, the O_f2 method, the O_f∞ method and the O_O method using the PPGs based on the individual subjects as well as the O_O method using the PPGs based on all the subjects. Figure 8 shows the order pairs of the estimated blood glucose values and the actual blood glucose values falling in the various regions in the Clarke error grid yielded by the various methods. Table 5 shows the percentages of these order pairs falling in the various regions in the Clarke error grid [33] yielded by different methods. It can be seen from Figure 8 that the O_f1 method, the O_f2 method and the O_f∞ method yield the best estimation performance in the low blood glucose range, the normal blood glucose range and the high blood glucose range, respectively. On the other hand, since the fusion method can enjoy the advantages of the individual methods, it can be seen from Table 4 and Table 5 that the fusion method yields the overall best performance. This demonstrates the advantage of using the fusion method. Moreover, since the individual modelling approach can eliminate the effects of different subjects having different responses on the infrared light, it can be seen from Table 4 and Table 5 that the O_O method using the PPGs from the individuals yields the better performance than those using the PPGs from all the subjects. This demonstrates the advantage of using the individual modelling approach.

**TABLE 4:** The values of R, mean absolute error (MAE), root mean squares error (RMSE) and MARD yielded by the various methods.

Methods	R	MAE±SD (mmol/L)	RMSE (mmol/L)	MARD
Fusion (PPGs based on the individuals)	**0.9466**	**0.6715 ± 1.8887**	**1.1068**	**0.0930**
O_f1 (PPGs based on the individuals)	0.8959	0.9581 ± 1.4041	1.1610	0.1259
O_f2 (PPGs based on the individuals)	0.8743	0.8889 ± 1.5229	1.1509	0.1223
O_f∞ (PPGs based on the individuals)	0.9103	0.7991 ± 1.8746	1.1319	0.1101
O_O (PPGs based on the individuals)	0.8515	0.9388 ± 1.4757	1.1631	0.1252
O_O (PPGs based on all the subjects)	0.8277	0.9869 ± 1.4028	1.1701	0.1471

*Note*: The bold values refer to the values corresponding to the best results.

FIGURE 8The order pairs of the estimated blood glucose values and the actual blood glucose values falling in the various regions in the Clarke error grid yielded by the various methods. (a) The fusion method using the PPGs based on the individuals. (b) The O_f1 method using the PPGs based on the individuals. (c) The O_f2 method using the PPGs based on the individuals. (d) The O_f∞ method using the PPGs based on the individuals. (e) The O_O method using the PPGs based on the individuals. (f) The O_O method using the PPGs based on all the subjects.

**Figure:**
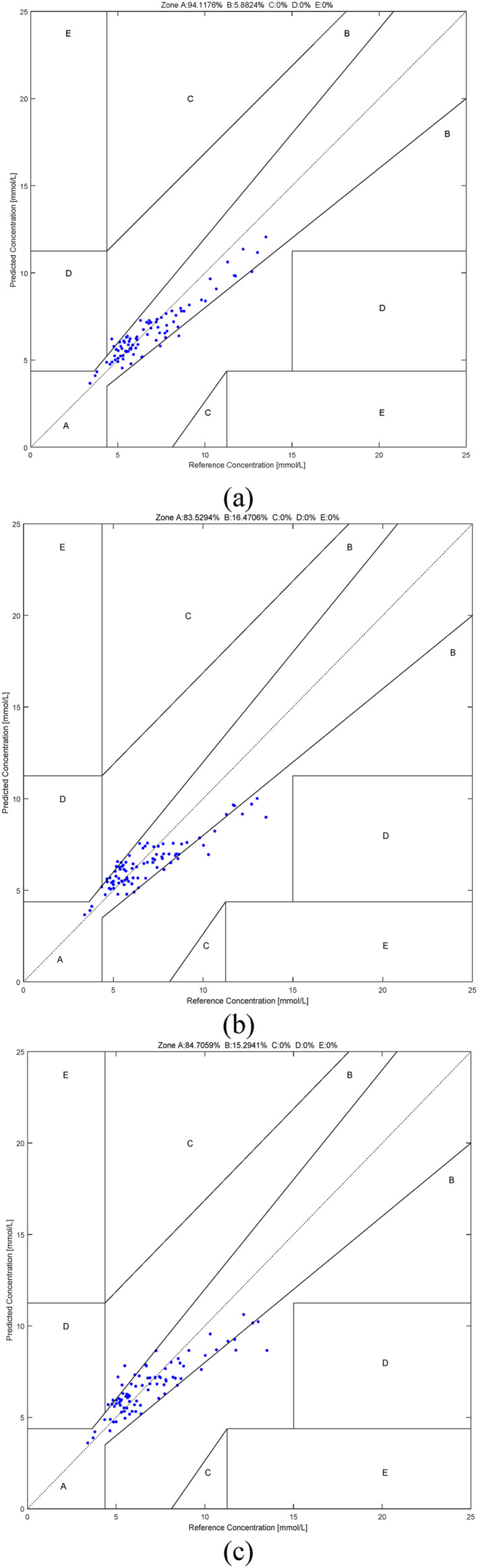

**Figure:**
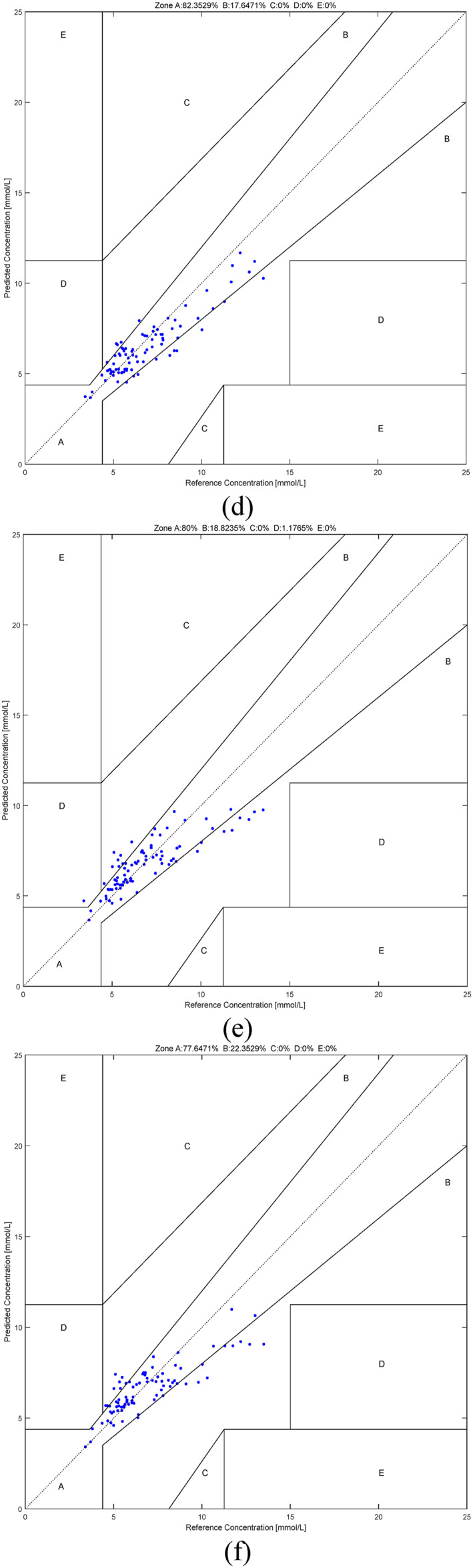

**TABLE 5:** The percentages of the order pairs of the estimated blood glucose values and the actual blood glucose values falling in the various regions in the Clarke error grid yielded by the various methods.

Methods	A	B	C	D	E
Fusion (PPGs based on the individuals)	**94.1176%**	5.882%	0%	0%	0%
O_f1 (PPGs based on the individuals)	83.5294%	16.4706%	0%	0%	0%
O_f2 (PPGs based on the individuals)	84.7059%	15.2941%	0%	0%	0%
O_f∞ (PPGs based on the individuals)	82.352%	17.6471%	0%	0%	0%
O_O (PPGs based on the individuals)	80%	18.8235%	0%	0%	0%
O_O (PPGs based on all the subjects)	77.647%	22.3529%	0%	0%	0%

*Note*: The bold values refer to the values corresponding to the best results.

### 3.2 Comparison to the existing methods

In order to have a fair comparison, the results yielded by all the methods are based on the same dataset established in this paper. Moreover, in order to verify the effectiveness of our proposed method, the results yielded by our proposed method are compared to those yielded by the current state of art method. In particular, since the SG filtering based method can effectively extract the underlying trend of the data and it is also based on the polynomial fitting approach, it is widely used for smoothing the features. Hence, this method is employed for performing the comparison. Here, the SG filtering with the following models including the RF model, the back propagation neural network (BPNN) model and the gradient boosting decision tree (GBDT) model is compared. It computes the total error within the window and the weights of the polynomial are obtained by minimising the total error via the least squares approach. Here, the window length is set at 5 and the order of the polynomial is set at 3. Table 6 shows the obtained results. Besides, the existing work which is the closest to our proposed method [34] is also compared. Table 7 shows the obtained results. It can be seen from Table 6 that our proposed method outperforms the SG method for all these three models. Also, it can be seen from Table 7 that our proposed method is superior to the existing work [34]. In particular, our proposed method can achieve the value of R at 0.9466, the MAE at 0.6715, the RMSE at 1.1068, the MARD at 0.0930 and the percentage of the test data falling in the region A of the Clarke error grid at 94.1176%. Moreover, the total time required for performing both the training and the testing based on our proposed algorithm is low.

**TABLE 6:** The values of R, mean absolute error (MAE), root mean squares error (RMSE) and MARD, the percentages of the test data points falling in the region A and the region B of the Clarke error grid as well as the total time required for performing both the training and the testing yielded by our proposed method and the Savitzky Golay (SG) smoothing method with different models.

Methods	R	MAE ± SD (mmol/L)	RMSE (mmol/L)	MARD	Percentages of the test data points falling in the region A and the region B of the Clarke error grid	Total time required for performing both the training and the testing (s)
Fusion of the various optimisation methods	**0.9466**	**0.6715 ± 1.8887**	**1.1068**	**0.0930**	**94.1176% (region A) 5.8824% (region B)**	0.3572s
SG with the RF model	0.7326	0.9676 ± 1.9917	1.1754	0.1284	84.6154% (region A) 15.3846% (region B)	0.2794s
SG with the BPNN model	0.4565	1.3728 ± 2.4569	1.2093	0.1890	67.3077% (region A) 32.6923% (region B)	0.6971s
SG with the GBDT model	0.6213	1.0876 ± 2.1365	1.1815	0.1432	82.6923% (region A) 17.3077% (region B)	**2.3045s**

*Note*: The bold values refer to the values corresponding to the best results.

**TABLE 7:** The values of R, mean absolute error (MAE), root mean squares error (RMSE) and MARD, the percentages of the test data points falling in the region A and the region B of the Clarke error grid as well as the total time required for performing both the training and the testing yielded by our proposed method and the existing work [34].

Methods	R	MAE±SD (mmol/L)	RMSE (mmol/L)	MARD	Percentages of the test data points falling in the region A and the region B of the Clarke error grid	Total time required for performing both the training and the testing (s)
Fusion of the various optimisation methods	**0.9466**	**0.6715 ± 1.8887**	**1.1068**	**0.0930**	**94.1176% (region A) 5.8824% (region B)**	**0.3572s**
Existing work [34]	0.8891	0.8761 ± 1.9708	1.2617	0.1188	87.0588% (region A) 12.9412% (region B)	4.6412s

*Note*: The bold values refer to the values corresponding to the best results.

## 4 CONCLUSION

For the non‐invasive blood glucose estimation, the LEDs emit the near infrared light and the near infrared light is received by the sensors for generating the PPGs. At the same time, the reference blood glucose values are taken. Then, the features are extracted from the PPGs. Next, the regression model is built based on the extracted features and the reference blood glucose values. Finally, for a given test data, the blood glucose value is estimated using the built model. However, due to the uncertainty of the acquisition device, the presence of the noise, the variations of the acquisition environments and different subjects having different responses of the infrared light to the blood glucose, the obtained features and the reference blood glucose values are highly unreliable. To address this issue, this paper proposes a polynomial fitting approach to smooth the obtained features or the reference blood glucose values to improve their reliability. In particular, the design of the weights in the polynomial is formulated as the various optimisation problems with the objective functions of different optimisation problems being the error function having different the norm criteria. Then, the results yielded by the various methods are fused together. Here, the training sets and the test sets of the regression models are defined based on the individual subjects. The computer numerical simulation results show that our proposed method yields 94.1176% of the data points falling in the region A of the Clarke error grid and the MARD at 0.0930.

Since the models are built by the individuals, the limitation of our proposed method is on the change of the physiological properties of the individuals such as the change of the medications taken by the individuals or the change of their meal habits. In this case, the correlation between the training set and the test set will be small. Like most of the machine learning algorithms, the estimation results will be poor. In future, the relationships among the individual models and the transfer from one individual model to another individual model will be investigated. Besides, the method will be developed for other biomedical signal processing applications such as the non‐invasive blood lipid estimation.

## AUTHOR CONTRIBUTIONS

Yiting Wei is responsible for developing the methodology, implementing the algorithm, acquiring the data and writing the draft of the paper. Bingo Wing‐Kuen Ling is responsible for attracting the funding, managing the project, developing the methodology, supervising the project and revising the paper. Danni Chen, Yuheng Dai and Qing Liu are responsible for validating the results and acquiring the data.

## CONFLICT OF INTEREST STATEMENT

There is no conflict of interest.

## Data Availability

The data will be available based on the request.

## References

[1] Sun, H. , et al.: IDF Diabetes Atlas: Global, Regional and Country‐Level Diabetes Prevalence Estimates for 2021 and Projections for 2045 (2021) 10.1016/j.diabres.2021.109119 PMC11057359 34879977

[2] Batmani, Y. : Blood glucose concentration control for type 1 diabetic patients: a non‐linear suboptimal approach. IET Syst. Biol. 11(4), 119–125 (2017). 10.1049/iet-syb.2016.0044 28721941 PMC8687382

[3] Zhang, G. , et al.: A noninvasive blood glucose monitoring system based on smartphone PPG signal processing and machine learning. IEEE Trans. Ind. Inf. 16(11), 7209–7218 (2020). 10.1109/tii.2020.2975222

[4] Li, Q.B. , et al.: Application of digital fourier filtering pretreatment method to improving robustness of multivariate calibration model in near infrared spectroscopy. Spectrosc. Spectr. Anal. 27(8), 1484–1488 (2007)

[5] Owen‐Reece, H. , et al.: Near infrared spectroscopy. Br. J. Anaesth. 82(3), 418–426 (1999). 10.1093/bja/82.3.418 10434827

[6] Nijboer, J.A. , Dorlas, J.C. , Mahieu, H.F. : Photoelectric plethysmography‐some fundamental aspects of the reflection and transmission methods. Clin. Phys. Physiol. Meas. 2(3), 205–215 (1981). 10.1088/0143-0815/2/3/004 7338024

[7] Arazuri, S. , Jarén, C. , Arana, J.I. : Selection of the temperature in the sugar content determination of kiwi fruit. Int. J. Infrared Millimet. Waves 26(4), 607–616 (2005). 10.1007/s10762-005-4076-8

[8] Cjarén, C. , et al.: White asparagus harvest date discrimination using NIRS technology. Int. J. Infrared Millimet. Waves 27(3), 391–401 (2006). 10.1007/s10762-006-9076-9

[9] Zhang, G.J. , et al.: Application of denoising and background elimination based on wavelet transform to blood glucose noninvasive measurement of near infrared spectroscopy. J. Infrared Millim. Waves 28(2), 107–110 (2009). 10.3724/sp.j.1010.2009.00107

[10] Li, L.N. , Zhang, G.J. , Li, Q.B. : Pretreatment method research of spectra in blood component non‐invasive measurement. Mod. Phys. Lett. B 23(07), 925–937 (2009). 10.1142/s0217984909019065

[11] Li‐Na, L. , Qing‐Bo, L. , Guang‐Jun, Z. : A wavelength selection method based on simple‐to‐use interactive self‐modeling mixture analysis for near infrared spectroscopy. Chin. J. Anal. Chem. 37(6), 823–827 (2009)

[12] Lu, H. , Zhang, G. : Analysis of system errors and accurate levelling. J. Zhejiang Univ. Technol. 29(4), 402 (2001)

[13] Sarker, S. , et al.: High nonlinearity and ultra high birefringence silicon core photonic crystal fiber. In: 2021 IEEE International Conference on Telecommunications and Photonics (ICTP), pp. 1–5. IEEE (2021)

[14] Sarker, S. , et al.: D‐shape photonic crystal fiber for optical coherence tomography: design and analysis. Opt. Eng. 60(12), 127109 (2021). 10.1117/1.oe.60.12.127109

[15] Sarker, S. , Arefin, M.A. , Islam, M.K. : Design and FEM analysis of a novel steering shaped photonic crystal fiber. In: 2021 5th International Conference on Electrical Information and Communication Technology (EICT), pp. 1–4. IEEE (2021)

[16] Arefin, M.A. , et al.: Analysis of reliable solutions to the boundary value problems by using shooting method. Math. Probl Eng. 2022, 2022–9 (2022). 10.1155/2022/2895023

[17] Albu, F. , Liu, J. , Grant, S.L. : Approximated proportionate affine projection algorithms for block‐sparse identification. In: 2016 13th International Conference on Electrical Engineering/Electronics, Computer, Telecommunications and Information Technology (ECTI‐CON), pp. 1–4. IEEE (2016)

[18] Albu, F. , Liu, J. , Grant, S.L. : A fast filtering block‐sparse proportionate affine projection sign algorithm. In: 2016 International Conference on Communications (COMM), pp. 29–32. IEEE (2016)

[19] Li, G. , et al.: The research status and development of noninvasive glucose optical measurements. Spectrosc. Spectr. Anal. 30(10), 2744–2747 (2010) 21137412

[20] Wang, W. , Bian, Z. , Zhang, D. : A model study on noninvasive blood glucose measurement with multi‐wavelength infrared array. J. Biomed. Eng. 20(4), 716–719 (2003) 14716885

[21] Liu, R. , et al.: Next step of non‐invasive glucose monitor by NIR technique from the well controlled measuring condition and results. Opt. Quant. Electron. 37(13), 1305–1317 (2005). 10.1007/s11082-005-4201-x

[22] Bernardi, L. , et al.: Heart rate‐respiration relationship: computerized method for early assessment of cardiac autonomic damage in diabetic patients. Acta Cardiol. 41(3), 197–206 (1986) 3490086

[23] Zhang, W.X. , et al.: Diabetic autonomic neuropathy in BB rats and effect of ARI treatment on heart‐rate variability and vagus nerve structure. Diabetes 39(5), 613–618 (1990). 10.2337/diabetes.39.5.613 2110085

[24] Scheff, J.D. , et al.: On heart rate variability and autonomic activity in homeostasis and in systemic inflammation. Math. Biosci. 252, 36–44 (2014). 10.1016/j.mbs.2014.03.010 24680646 PMC4159048

[25] Barbieri, R. , et al.: A point‐process model of human heartbeat intervals: new definitions of heart rate and heart rate variability. Am. J. Physiol. Heart Circ. Physiol. 288(1), H424–H435 (2005). 10.1152/ajpheart.00482.2003 15374824

[26] Tavassoli, M. , Ebadzadeh, M.M. , Malek, H. : Classification of cardiac arrhythmia with respect to ECG and HRV signal by genetic programming. CJAIMLPR 3(1), 1–8 (2012)

[27] Amanipour, R. , et al.: The effects of blood glucose changes on frequency‐domain measures of HRV signal in type 1 diabetes. CONIELECOMP, 50–54 (2012). IEEE

[28] Kamal, A.A.R. , et al.: Skin photoplethysmography—a review. Comput. Methods Progr. Biomed. 28(4), 257–269 (1989). 10.1016/0169-2607(89)90159-4 2649304

[29] Stein, P.K. , Kleiger, R.E. , Rottman, J.N. : Differing effects of age on heart rate variability in men and women. Am. J. Cardiol. 80(3), 302–305 (1997). 10.1016/s0002-9149(97)00350-0 9264423

[30] LinWuLi, W.H.D.C. , Zhang, H. , Zhang, Y.T. : Comparison of heart rate variability from PPG with that from ECG. IFMBE Proc., 213–215 (2014). 10.1007/978-3-319-03005-0_54

[31] Nardelli, M. , et al.: Assessing the quality of heart rate variability estimated from wrist and finger ppg: a novel approach based on cross‐mapping method. Sensors 20(11), 3156 (2020). 10.3390/s20113156 32498403 PMC7309104

[32] Monte‐Moreno, E. : Non‐invasive estimate of blood glucose and blood pressure from a photoplethysmograph by means of machine learning techniques. Artif. Intell. Med. 53(2), 127–138 (2011). 10.1016/j.artmed.2011.05.001 21696930

[33] Clarke, W.L. , et al.: Evaluating clinical accuracy of systems for self‐monitoring of blood glucose. Diabetes Care 10(5), 622–628 (1987). 10.2337/diacare.10.5.622 3677983

[34] Zhou, X. , et al.: Joint empirical mode decomposition, exponential function estimation and L 1 norm approach for estimating mean value of photoplethysmogram and blood glucose level. IET Signal Process. 14(9), 652–665 (2020). 10.1049/iet-spr.2020.0096

